# Cognitive training and brain stimulation in prodromal Alzheimer’s disease (AD-Stim)—study protocol for a double-blind randomized controlled phase IIb (monocenter) trial

**DOI:** 10.1186/s13195-020-00692-5

**Published:** 2020-11-07

**Authors:** Friederike Thams, Anna Kuzmina, Malte Backhaus, Shu-Chen Li, Ulrike Grittner, Daria Antonenko, Agnes Flöel

**Affiliations:** 1 Department of Neurology, Universitätsmedizin Greifswald, Ferdinand-Sauerbruch-Straße, 17475 Greifswald, Germany; 2 Chair of Lifespan Developmental Neuroscience, Faculty of Psychology, TU Dresden, Zellescher Weg 17, 01062 Dresden, Germany; 3 Centre for Tactile Internet with Human-in-the-Loop, TU Dresden, 01062 Dresden, Germany; 4 Berlin Institute of Health (BIH), Anna-Louisa-Karsch-Straße 2, 10178 Berlin, Germany; 5 Charité – Universitätsmedizin Berlin, Corporate Member of Freie Universität Berlin, Humboldt-Universität zu Berlin, And Berlin Institute of Health, Institute of Biometry and Clinical Epidemiology, Charitéplatz 1, 10117 Berlin, Germany; 6 German Centre for Neurodegenerative Diseases (DZNE) Standort Greifswald, Greifswald, Germany

**Keywords:** Transcranial direct current stimulation, Aging, Subjective cognitive decline, Mild cognitive impairment, Working memory, Decision-making, Transfer

## Abstract

**Background:**

Given the growing older population worldwide, and the associated increase in age-related diseases, such as Alzheimer’s disease (AD), investigating non-invasive methods to ameliorate or even prevent cognitive decline in prodromal AD is highly relevant. Previous studies suggest transcranial direct current stimulation (tDCS) to be an effective method to boost cognitive performance, especially when applied in combination with cognitive training in healthy older adults. So far, no studies combining tDCS concurrent with an intense multi-session cognitive training in prodromal AD populations have been conducted.

**Methods:**

The AD-Stim trial is a monocentric, randomized, double-blind, placebo-controlled study, including a 3-week tDCS-assisted cognitive training with anodal tDCS over left DLPFC (target intervention), compared to cognitive training plus sham (control intervention). The cognitive training encompasses a letter updating task and a three-stage Markov decision-making task. Forty-six participants with subjective cognitive decline (SCD) or mild cognitive impairment (MCI) will be randomized block-wise to either target or control intervention group and participate in nine interventional visits with additional pre- and post-intervention assessments. Performance in the letter updating task after training and anodal tDCS compared to sham stimulation will be analyzed as primary outcome. Further, performance on the second training task and transfer tasks will be investigated. Two follow-up visits (at 1 and 7 months post-training) will be performed to assess possible maintenance effects. Structural and functional magnetic resonance imaging (MRI) will be applied before the intervention and at the 7-month follow-up to identify possible neural predictors for successful intervention.

**Significance:**

With this trial, we aim to provide evidence for tDCS-induced improvements of multi-session cognitive training in participants with SCD and MCI. An improved understanding of tDCS effects on cognitive training performance and neural predictors may help to develop novel approaches to counteract cognitive decline in participants with prodromal AD.

**Trial registration:**

ClinicalTrials.gov, NCT04265378. Registered on 07 February 2020. Retrospectively registered.

Protocol version: Based on BB 004/18 version 1.2 (May 17, 2019).

Sponsor: University Medicine Greifswald.

## Background

Prodromal Alzheimer’s disease (AD) starts several years before the clinical diagnosis of dementia and can be subdivided into at least two stages. Participants with subjective cognitive decline (SCD) experience cognitive impairments, which are not yet evident in neuropsychological measures [1]. In participants with mild cognitive impairment (MCI), subjective cognitive decline as well as first objective cognitive impairments on neuropsychological testing are evident. Application of non-pharmacological therapeutic interventions during these prodromal stages of AD may halt or at least decelerate the neurodegenerative progress, thus preserve clinically unobtrusive stages for as long as possible [2, 3]. Previous studies investigating single session non-invasive brain stimulation (NIBS) influence on cognitive task performance have shown beneficial effects on cognition in healthy older adults [4] as well as in SCD [5] and MCI [6–8]. NIBS may ameliorate brain network deficiencies [7] and possibly delay the neuropathological disease progression by increasing the release of brain-derived neurotrophic factor or boosting β-amyloid clearance from the brain [9, 10].

Multi-session study designs, implementing concurrent application of transcranial direct current stimulation (tDCS) and multi-day cognitive training, yielded promising results with regard to improved performance in samples of healthy older adults [11–14]. Anodal tDCS is thought to facilitate cortical excitability by changing the resting membrane potential towards depolarization [15, 16]. Immediate effects influence voltage-dependent ion channels, whereas longer stimulation is thought to elicit long-term potentiation mechanisms [17]. These alterations are particularly effective in already activated functionally connected regions. tDCS may therefore support the effects of cognitive training by facilitating ongoing cognitive processes [9, 18]. Evidence with regard to sustained benefits of the intervention or transfer to non-trained cognitive domains has not been unequivocal so far [19–22].

In the AD-Stim study, we will assess in a double-blind randomized controlled phase IIb clinical trial if such a combined multi-session training plus tDCS intervention yields substantial long-term benefits and transfer effects in a prodromal AD population.

We will administer a multi-session combined cognitive training and tDCS intervention in participants with prodromal AD (*N* = 46). Half of the sample will receive anodal tDCS over the left dorsolateral prefrontal cortex (DLPFC) while performing the cognitive training, whereas the other half will undergo sham stimulation during training. Left DLPFC was chosen as a stimulation target because of its involvement in executive functions [23]. Moreover, left DLPFC has been shown to be under-recruited during the performance of executive tasks in older compared to younger adults [24]. Previous studies using anodal tDCS over this area demonstrated beneficial effects on executive performance in non-clinical populations [13, 25, 26]. We will allocate participants to anodal and sham tDCS groups using stratified block randomization with a 1:1 ratio (strata: age and baseline performance in the trained updating task). We will assess behavioral outcome measures, such as direct training effects, transfer to non-trained domains, and long-term effects at multiple time points. We will further elucidate neural predictors of interventional success before the intervention and assess neural correlates at long term. This protocol, describing the design and methods of the AD-Stim study, was prepared in accordance with the SPIRIT guidelines [27, 28].

## Methods: participants, intervention, and outcomes

### Design and setting

**Fig. 1:**
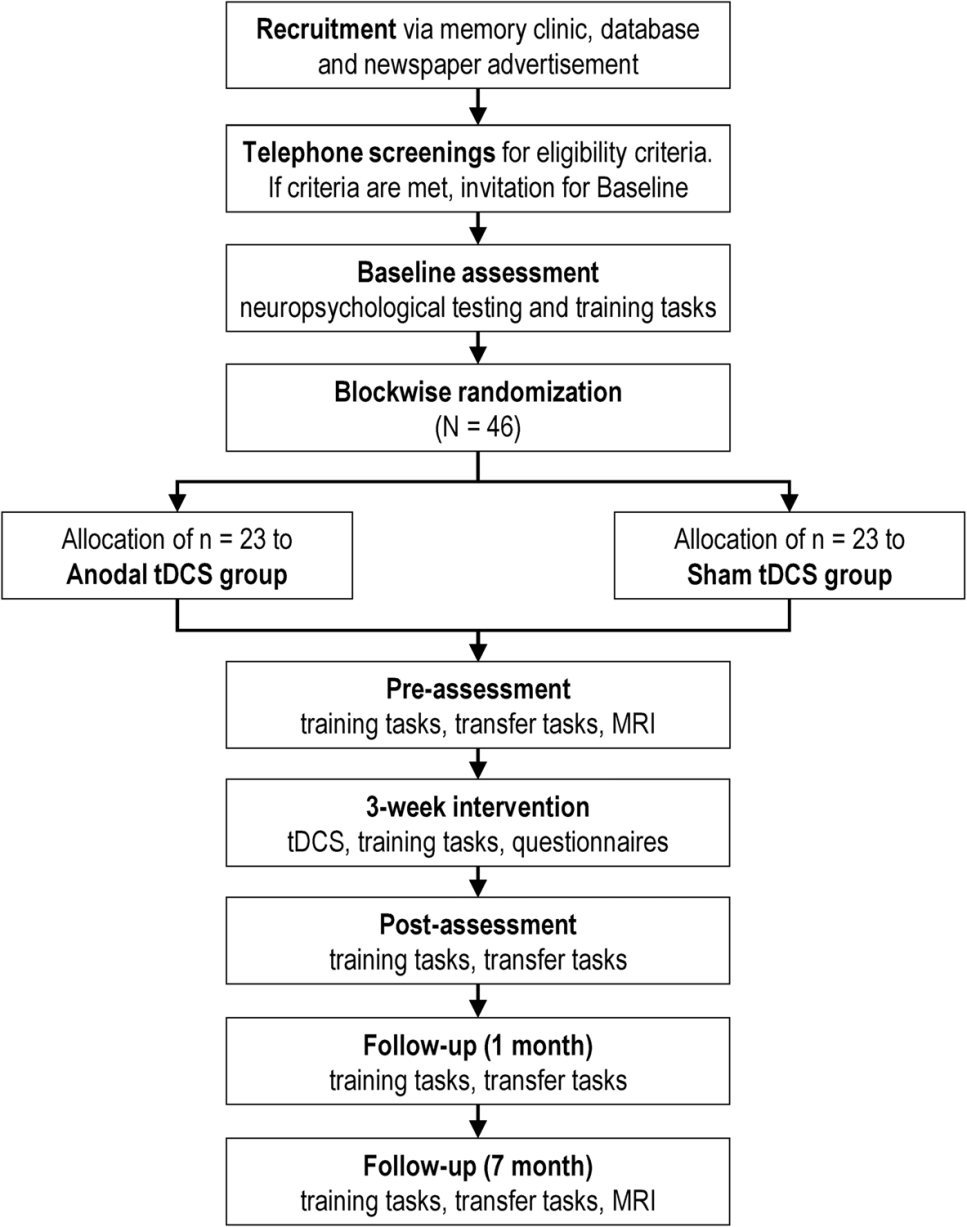
AD-Stim study flowchart. tDCS, transcranial direct current stimulation; MRI, magnetic resonance imaging

This is a monocentric, randomized, double-blind, placebo-controlled study, including a 3-week electrical brain stimulation-assisted cognitive training with anodal tDCS over the left DLPFC, compared to cognitive training plus sham, a study design similar to our current trial in healthy older adults [29]. Participants with prodromal AD will participate in nine interventional visits with additional pre- and post-intervention visits, taking place at University Medicine Greifswald. Two follow-up visits (at 1 and 7 months post-training) will be performed to also assess possible maintenance effects. Magnetic resonance imaging (MRI) will be performed before the intervention and at the 7-month follow-up. A flowchart of the study is shown in Fig. 1.

### Eligibility criteria

Before randomization, participants eligible for the study must meet all the following criteria:

1. Age, 60–80 years
2. Right-handedness
3. Presence of either SCD or MCI as defined by self-perceived cognitive decline, unrelated to an acute event and persistent over at least 6 months; worrying about this decline and report of having attended or being willing to attend a physician about it and performance in neuropsychological screening at baseline either within (SCD) or below (MCI) normal range (normal range defined as performance on each subtest within − 1.5 SD from the normative sample’s mean) [1, 30].
  Additionally, alternative etiologies of cognitive decline will be excluded by medical history or, if appropriate, by serum analyses (metabolic, inflammatory, and vitamin deficiency), and cerebral MRI (brain tumor, stroke), and questionnaires on quality and duration of sleep, and current medication (severe sleep disturbances, current medication interfering with cognitive performance, including sedative and psychotropic medication).

In case one or more of the following criteria are present at randomization, potential participants will be excluded:

1. Dementia or other neurodegenerative neurological disorders, epilepsy or history of seizures, close relatives with epilepsy or history of seizures, and previous stroke
2. Severe and untreated medical conditions that preclude participation in the training, as determined by the responsible physician
3. History of severe alcoholism or use of drugs
4. Severe psychiatric disorders such as psychosis or depression (if not in remission).

Note that contraindication to MRI will not be treated as exclusion criteria as participants will still be included in the study sample, but no MRI scans will be performed in these individuals. Further, smoking will not be an exclusion criterion. Participants will however be instructed to follow their usual smoking habits to avoid deprivation-induced plasticity decline [31, 32], which will be assessed by questionnaires at each session. If all eligibility criteria are met and participants provide written informed consent, they will be included in the study sample.

### Intervention

**Fig. 2:**
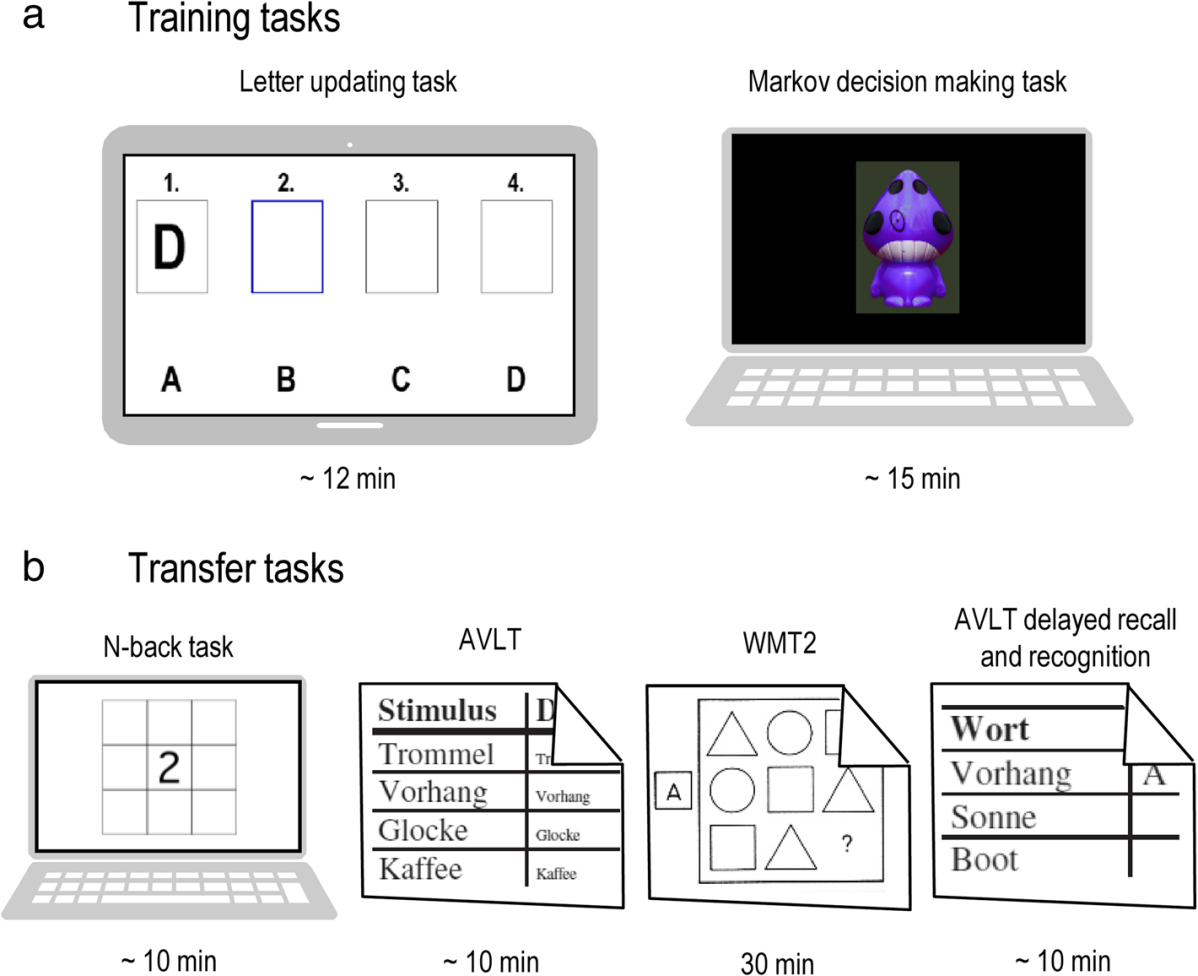
Task overview. **a** Training tasks performed at each visit. **b** Transfer tasks performed at pre-, post-, and follow-up assessments. AVLT, auditory verbal learning test [33]; WMT-2, Wiener matrices test [34]

Each of the nine training visits comprises two cognitive training tasks (Fig. 2), being performed by the participants, concurrently receiving either anodal or sham tDCS. Before beginning with the training, the stimulation set-up will be mounted.

The first training task will be a letter updating task (cf. [19]), presented on a tablet computer, and training updating of information stored in working memory. Lists of letters A to D (with lengths of 5, 7, 9, 11, or 13 letters; three times each; a total of 15 lists) will be presented in random order, one letter at a time (presentation duration 2000 ms, inter-stimulus interval 500 ms). After each list, participants will have to recall the last four letters that were presented.

Second, participants will be presented with a three-stage Markov decision-making task on a laptop computer (cf. [24, 35, 36]), where they have to choose between two actions, i.e., pressure of left or right key, which results in an action-related reward (monetary gain or loss). The underlying Markov probability defines that a decision at a given state determines not only the reward, but also the transition into the next out of three decisional stages. This requires the participants to learn choosing the optimal sequence of action to maximize overall gains, minimize overall losses, and thus continuously transition through all three stages. There will be two different learning conditions. In the *immediate reward condition*, the action-outcome associations will be equal for all three stages. Here, the optimal choice is always associated with a gain (+ €0.05), whereas choosing the other alternative results in a loss (− €0.05). In the *delayed reward condition*, the action-outcome associations will vary over the three stages. An optimal action choice will be related to a small loss (− €0.05) in the first two stages and a larger gain (+ €0.25) in the third stage. A chain of suboptimal action choices however will result in a small gain (+ €0.05) in the first two stages and a large loss (− €0.25) in the third stage.

TDCS will be delivered using a neuroConn DC-Stimulator Plus (neuroCARE Group GmbH, Munich, Germany). Direct current will be delivered via two rectangular saline-soaked synthetic sponge electrodes (size 5 × 7 cm) connected to the stimulator and centered over the left DLPFC (anode, F3) with the longitudinal edge horizontally aligned and right supraorbital cortex (cathode, Fp2) with the transverse edge horizontally aligned. The anodal tDCS group will receive stimulation for 20 min and the sham tDCS group will receive stimulation for 30 s to elicit similar tingling sensations and blind participants for stimulation conditions (current intensity 1 mA, 10 s fade in and out) [37, 38].

Stimulation will be started simultaneously with the first training task. Participants will be instructed to avoid excessive alcohol consumption or smoking on the day of the study, to adhere to their usual sleep duration, and to avoid drinking caffeine 90 min prior to the training visits. Adverse events will be assessed via a questionnaire every third training visit [39].

### Outcome measures

At each visit, outcome measures for the training tasks will be acquired. Additional outcomes for possible transfer effects will be acquired at pre-, post-, and follow-up assessments (see Fig. 2). Table 1 displays all assessment time points and acquired measures. Analyses for each measure will compare potential differences between anodal and sham tDCS groups (Table 1).

**Table 1:**
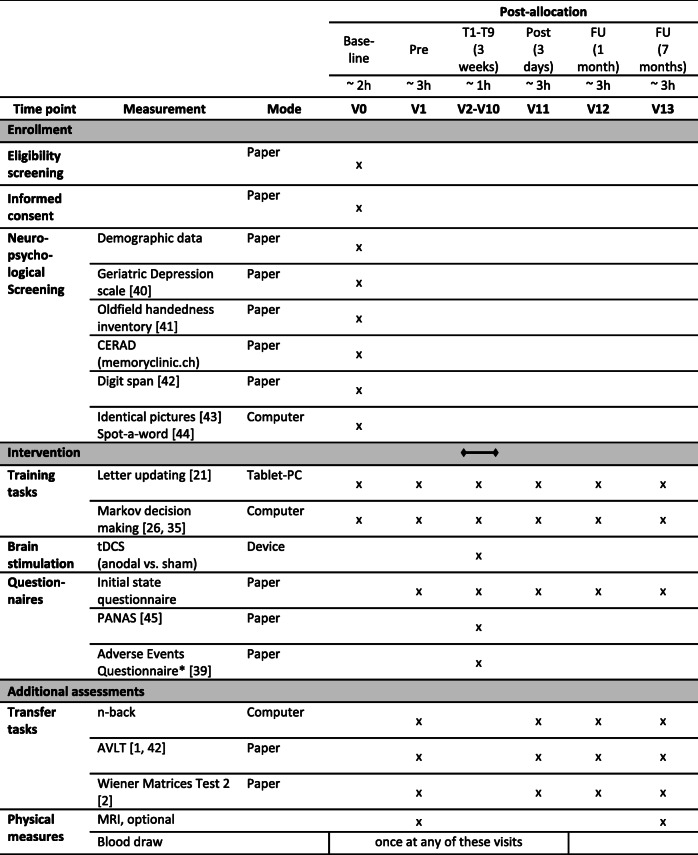
AD-Stim outcome measures

Abbreviations: *T1–T9* training 1–9; *FU* follow-up assessment; *V0–V13* visits 0–13; *CERAD* The Consortium to Establish a Registry for Alzheimer’s Disease, neuropsychological battery; *Fragebogen zur Ausgangslage* questionnaire about the current state; *PANAS* positive and negative affect schedule [45]; *AVLT* German version of the auditory verbal learning test; *tDCS* transcranial direct current stimulation; MRI magnetic resonance imaging. All measures were acquired on site, except for screening, which was done via telephone. *Assessed only at the end of each training week (V4, 7, and 10)

#### Primary outcomes

The primary outcome measure will be working memory performance at post-assessment, as measured by the number of correctly recalled lists in the letter updating task.

#### Secondary outcomes

The secondary outcome will be performance in decision-based learning at post-assessment, operationalized by the proportion of optimal actions in the Markov decision-making task.

Additionally, for the main measures of the training tasks (number of correctly recalled lists in the letter updating task and proportion of optimal actions in the Markov decision-making task), analyses of performance at follow-up assessments will be conducted and learning curves as well as online and offline effects of the intervention, i.e., within-session performance and performance changes from the last trial of the previous visit to the first trial of the next visit, will be assessed.

Further secondary outcomes will be assessed at pre-, post-, and follow-up assessments and include:

- *Transfer tasks* encompassing working memory performance, as assessed by performance in a numeric n-back task (% correct, d-prime); episodic memory performance, as measured by performance in the German version of the auditory verbal learning test [33, 42] (total number of words learned, number of recalled words at delayed recall); and reasoning ability, as assessed by the Wiener matrices test (WMT2) [34] (% correct). All transfer measures will be corrected for performance at pre-assessment.
- Structural and functional *neural correlates* (assessed at pre- and 7-month follow-up assessments), as measured by structural and functional MRI.

#### Exploratory analyses

To assess outcomes of the two training tasks in more detail, exploratory analyses of further measures of the two training tasks will be conducted (e.g., outcomes dependent on list length in the letter updating task, parameters from a drift diffusion model for the Markov decision-making task). Moreover, to identify characteristics associated with training and tDCS effects, measures of cognitive reserve (e.g., education, baseline cognitive ability, or neuropsychological status) will be analyzed. Lastly, genetic polymorphisms such as ApoE, COMT, and BDNF, derived from the analysis of blood samples and related to cognition, will be included as potential modulators of response to tDCS [9].

### Participant timeline

Individuals will participate in 14 visits with two additional MRI sessions, taking place at the University Medicine Greifswald. After inclusion at the baseline visit (V0), participants will attend the pre-assessment visit (V1) before starting the nine training visits during three consecutive weeks on 3 days a week (V2–V10). After the training, post-assessment (V11) will be conducted, and 4 weeks later, a first follow-up visit (V12) will be administered; a second follow-up (V13) visit will be 7 months after post-visit. MRI will be acquired before pre-assessment (V1) and at the second follow-up (V13).

#### Baseline measures

After providing informed consent and participating in a demographic interview, depression screening and handedness questionnaire will be administered at the baseline visit (V0). We will then assess performance in various cognitive domains (Table 1). Furthermore, the two training tasks will be performed as described above, except that at baseline, the letter updating task starts with one practice trial with 4 lists. The baseline visit will take approximately 3 h.

#### Pre-, post-, and follow-up assessments

At pre-, post-, and follow-up visits, the investigator will perform a semi-structured interview on the self-reported well-being of the participant, quality and duration of sleep, and potential stressors 2 h prior to the visit. Then, the training and transfer tasks will be performed.

### Sample size

Power calculation is based on recent studies using multi-session application of anodal tDCS during cognitive training compared to training with sham tDCS on immediate performance in the trained task (primary outcome) [11, 46, 47]. Based on these data, we estimated an effect size of 0.85. To demonstrate an effect in the primary outcome (number of correctly recalled lists in the letter updating task) with an independent *t* test using a two-sided significance level of 0.05 and a power of 80%, 46 participants (23 per group) need to be included. This conservative approach using a *t* test was chosen, even though we intend to analyze the primary outcome conducting analysis of covariance (ANCOVA) models [48]. Sample size estimation was conducted using nQuery Advisor 8.5.1 [49].

### Recruitment

Participants will be recruited from neurological departments of local clinics and doctors’ offices as well as through newspaper advertisements in the local newspapers and distribution of flyers in local senior citizen clubs. Telephone screenings will be conducted with all potential participants, and study information will be provided. Eligible candidates will be invited for the baseline visit. Following the baseline visit (V0), participants will be included if eligibility criteria are met.

## Methods: assignment of interventions

Allocation to anodal and sham tDCS groups will be performed using stratified block randomization. Participants will be randomly allocated by a researcher not involved in assessments. Allocation to the experimental groups (anodal vs. sham) will be performed with a 1:1 ratio with age (two age strata) and baseline and performance in the letter updating task (two performance strata) as strata. We chose cut-offs of 70 years and 2 lists correct in the letter updating task. Randomization blocks with varying block sizes will be generated for each of the four groups, using R software (http://www.R-project.org) and the blockrand package (https://CRAN.R-project.org/package=blockrand). Participants will then be allocated to the anodal or sham tDCS group, based on the generated randomization sequences within each block and stratum.

### Blinding

This is a double-blind trial, where blinding of study assessors is ensured by using the study mode of the DC stimulator. The researcher performing randomization will provide the study assessors with a code per participant to be entered into the device to start the stimulation. Assessors will be unaware of whether the code starts anodal or sham tDCS. To blind participants to the experimental conditions, in the sham tDCS group, current will be applied for 30 s to elicit the typical tingling sensation of stimulation on the scalp. Previous research showed that sham tDCS is a safe and valid method of blinding study participants [37, 38]. After the last training visit, participants will be asked to state whether they believed they received anodal or sham tDCS.

## Methods: data collection, management, and analysis

### Data collection methods

Neuropsychological and behavioral data and blood samples will be collected from each participant. MRI data will be collected, unless there are contraindications to MRI scanning. Furthermore, we will collect information on cerebrospinal fluid (CSF) biomarkers (T-tau, P-tau_181_, Aβ1–42, or Aβ1–42/Aβ1–40) from clinical files, if available. Study assessors will be thoroughly trained in administering the assessments. In Table 1, time points of data collection are shown.

#### Neuropsychological and behavioral assessment

Neuropsychological testing at the baseline visit (V0) comprises paper-pencil as well as computer-based assessments. Geriatric Depression Scale [40] and the Edinburgh Handedness Inventory [41] will be administered. Performance in several cognitive domains will be tested with CERAD-Plus test battery (memoryclinic.ch, [50]), digit span test [42], identical pictures task [43], and spot-a-word task [44].

The two training tasks will be performed at every visit and are descripted in detail in the “Intervention” section. Encompassing paper-pencil and computer-based assessments, the transfer tasks will be administered at pre-, post-, and follow-up visits (V1, V11–13). First, participants will perform a numeric n-back task (1 and 2 back) and the German version of the auditory verbal learning test [33, 42]. Then, participants will perform the Wiener matrices test (WMT2) in the 30-min interval to assess long-term memory [34].

#### Magnetic resonance imaging

MRI will be acquired at the Baltic Imaging Center (Center for Diagnostic Radiology and Neuroradiology, Universitätsmedizin Greifswald) with a 3-Tesla scanner (Siemens Verio) using a 32-channel head coil, prior to the training intervention and at 7-month follow-up. A T1-weighted 3D sequence, a 3D FLAIR, a diffusion tensor imaging (DTI), and a resting-state fMRI sequence will be recorded; detailed information on all MRI sequences is provided in Table 2. Additional T1- and T2-weighted structural images will be acquired with parameters optimized for computational modeling to calculate electric field distributions (simnibs.org, [51, 52]). Seed-based resting-state functional connectivity within and between large-scale task-relevant networks (e.g., frontoparietal and default mode network) will be performed [11, 53, 54] using the CONN toolbox (www.nitrc.org/projects/conn, [55]). White-matter pathways will be reconstructed from diffusion-weighted images using TRACULA pipeline [56] in Freesurfer (http://surfer.nmr.mgh.harvard.edu/), in order to extract tract fractional anisotropy and mean diffusivity [57–60]. Changes in FA and MD on whole-brain level will be analyzed using FSL’s tract-based spatial statistics (TBSS, www.fmrib.ox.ac.uk/fsl, [61, 62]). MD in gray matter will also be explored for examination of intervention-induced microstructural gray matter change [63]. Segmentation on high-resolution T1 scans will be performed to assess the volume of cortical and subcortical gray matter [19, 64] using the computational anatomy toolbox (CAT12, http://www.neuro.unijena.de/cat/) and Freesurfer (http://surfer.nmr.mgh.harvard.edu/).

**Table 2:** Neuroimaging data acquisition parameters

Sequence	Main parameters
Resting-state fMRI	TR = 2000 ms, TE = 30 ms, FOV 192 × 192 mm^2^, 34 slices, 176 volumes, descending acquisition, 3.0 × 3.0 × 3.0 mm^3^, flip angle 90°
T1 MPRAGE	TR = 2300 ms, TE =2.96 ms, TI = 900 ms, 192 slices, 1.0 × 1.0 × 1.0 mm^3^, flip angle 9°
DTI	TR = 11,100 ms, TE = 107 ms, 70 slices, 1.8 × 1.8 × 2.0mm^3^, 64 directions (*b* = 1000 s/mm^2^)
FLAIR	TR = 5000 ms, TE = 388 ms, 160 slices, 1.0 × 1.0 × 1.0 mm^3^
T1w	TR = 1690 ms, TE = 2.52 ms, TI = 900 ms, 176 slices, 1.0 × 1.0 × 1.0 mm^3^, flip angle 9°, using selective water excitation for fat suppression
T2w	TR = 12,770 ms, TE = 86.0 ms, 96 slices, 1.0 × 1.0 × 1.0 mm^3^, flip angle 111°

*Abbreviations*: *TR* repetition time, *TE* echo time, *TI* inversion time, *FOV* field of view, *fMRI* functional magnetic resonance imaging, *DTI* diffusion tensor imaging, *FLAIR* fluid-attenuated inversion recovery, *MPRAGE* magnetization prepared rapid acquisition gradient echo

#### Blood draw

A blood sample for conducting genetic analyses will be collected from all participants, preprocessed, and stored at the Neuroimmunology Lab of the University Medicine Greifswald, using the cryo-preservation method. Having collected the full sample, genetic polymorphisms relevant for learning (ApoE, COMT, and BDNF) [65–68] will be analyzed at the Department of Psychiatry, Psychotherapy and Psychosomatic, University Medicine, Halle/Saale, Germany.

#### Retention and adherence

To ensure retention throughout the study, participants will be provided with information about their appointments via telephone. A letter with detailed study information and a printout of all study sessions will be sent to all participants. If applicable, the participants’ relatives will also be informed about the study and the upcoming appointments. Additionally, time and date of the next visit will be discussed at each visit. In case of not being able to attend a visit or wanting to reschedule, participants will be encouraged to leave a message on the study site’s 24/7 answering machine and will then be contacted by the study team. All study participants will receive a reasonable financial reimbursement (approximately 10 € per hour), the results of their neuropsychological screening, and, if they underwent MRI scanning, their structural MRI images on a compact disc.

Any effort to collect as much data as feasible from the participant will be made, if complete adherence to the protocol is not possible.

### Data management and monitoring

All participant data will be pseudonymized, and spreadsheets containing participant IDs as well as personal data will be secured with a password, solely known by study staff. Digital data, i.e., output files from computer-based tasks, will be stored on a secure file server directly after acquisition. Non-digitally acquired data will be manually digitalized by a member of the research staff and double-checked by another member. Progress of data entry and checking procedures will be documented. Files containing subject records will be sorted by participant ID for easy access and stored securely. Sensitive data, such as names and medical records, will be stored separately in a lockable cabinet. All digitally acquired data, e.g., output files from computer-based tasks, will be stored on a secure file server, and MRI data will be pseudonymized before analysis. Following good scientific practice, data will be stored for at least 10 years.

### Adverse event monitoring

The risk of health damage through anodal tDCS is expected to be minimal, and known adverse events (AEs) associated with the method and the study parameters (20 min, 1 mA) are restricted to tingling at the electrode sites, skin reddening under the electrode, and, less frequently, a mild headache [39]. Participants will be informed about all possible risks and about their right to withdraw consent at any time without providing cause. An adverse event questionnaire [39] will be implemented at the end of every third stimulation visit (V4, V7, V10), to monitor possible AEs at a reasonable frequency, without drawing the participant’s attention too much to stimulation-induced sensations and cause distractions from the tasks. Further, study assessors will monitor and document possible incidence of AEs and serious AEs (SAEs). In case an SAE occurs, the study physician will first make an assessment as to whether or not a causal relationship with the intervention is considered possible. If more than three of the enrolled participants suffer from SAEs that are likely to be associated with the intervention (as assessed by the study physician), the trial will be discontinued.

### Statistical analysis

Detailed analyses of primary and secondary outcomes will be reported in the statistical analysis plan to be written and registered before unblinding of investigators performing the analyses. Confirmatory analysis of treatment effects will be conducted within an intention to treat (ITT) framework with multiple imputed data sets in case of missing data (under the assumption of missing completely at random or missing at random). Further as sensitivity analyses, we will perform “per protocol” analyses, including only those participants who finished post-assessment.

Statistical analyses will be divided to analyze:

1. Immediate treatment effects by including all measures including V11 (post-assessment)
2. Long-term treatment effects by focusing on V12 (1-month follow-up) and V13 (7-month follow-up).

Using linear mixed models, the measures of the letter updating task over the study period including V11 (post-assessment) will be used as dependent variable, the stimulation group (tDCS, sham) as factor, and letter updating performance at pre-assessment as well as age as covariates. The primary outcome (letter updating task score at post-assessment) will be evaluated between treatment groups based on this regression model via marginal means. We will use random intercept models that account for the clustering of measures within individuals. Secondary outcomes, i.e., performance on the second training task (Markov decision-making task) and on the transfer tasks, will be analyzed using similar statistical models. All models will be corrected for performance at pre-assessment. Structural and functional neural data will be analyzed on the whole-brain level, using general linear models, implemented in the analysis software. To assess brain-behavior associations, correlations between behavioral and neuronal parameters will be calculated. In case of violation of requirements for parametric methods, data will be transformed before analysis or appropriate non-parametric tests will be conducted. Data analysis will be conducted using IBM SPSS Statistics for Windows (IBM Corp., Armonk, NY), MatLab (The Mathworks Inc., 2016), and R software (https://www.R-project.org).

## Ethics and dissemination

The study was approved by the ethics committee of the University Medicine Greifswald and will be conducted in accordance with the Helsinki Declaration. All data collected will be pseudonymized. The results of the study will be made accessible to scientific researchers and health care professionals via publications in peer-reviewed journals and presentations at national and international conferences. Furthermore, the scientific and lay public can access the study results on the ClinicalTrials.gov website (identifier: NCT04265378).

## Discussion

This randomized controlled double-blind trial will investigate the effects of a combined multi-session cognitive training and tDCS intervention in participants with SCD and MCI on trained and untrained cognitive functions. Anodal tDCS will be administered over the left DLPFC (with the cathode over the contralateral supraorbital cortex) while participants perform two consecutive cognitive training tasks. The control group will receive sham tDCS during performance on the same tasks.

In sum, we will elucidate the cognitive impact and its underlying mechanisms and determinants of combined training and tDCS effects. Thus, the results of this trial will contribute to developing novel interventions for the improvement of cognitive functions in prodromal AD.

This trial will include a sample of well-characterized older adults with MCI or SCD as carefully characterized by clinical assessments, structured interviews, questionnaires, and neuropsychological testing at baseline visit. Available information on CSF biomarkers will be retrieved from clinical files to complement behavioral assessments. In order to be able to draw firm conclusions about the effectiveness of the intervention, participants will be randomized into anodal and sham tDCS groups, stratified by age and initial performance on the working memory training task. Consequently, both interventional groups will be comparable for baseline performance and age. Further, as cognitive impairments in prodromal AD progress over time [69] and tDCS effects may not be evident directly after stimulation, but only on long-term follow-up [47, 70], participants will be invited for follow-up visits at 4 weeks and 7 months after the intervention. These long-term assessments might clarify the differential long-term intervention effects between groups, potentially showing less decrease in cognitive performance in the anodal group compared to sham. Such a finding would indicate the potential of training plus tDCS to halt the progression of cognitive decline in participants with SCD and MCI.

The cognitive training was designed to target executive functions, specifically working memory and decision-making abilities, which, when preserved and intact, allow for coherent goal-directed actions and are highly relevant for activities of daily living [71–73]. Executive functions are known to be modulated by the prefrontal cortex [72, 74, 75] and to be affected early on in prodromal AD [69]. These early stages and AD itself constitute several challenges for affected participants and their personal environment as well as for society and health care systems. It is therefore of high clinical interest to investigate effective interventions against the decline in these cognitive domains. The growing proportion of older adults calls for suitable interventions for age-associated diseases such as AD. From the scientific perspective, this research contributes to the understanding of neural mechanisms underlying cognitive processes in older adults [76–78].

## Conclusion

In summary, with this phase IIb clinical trial, we will elucidate the immediate and long-term effects of a 3-week combined cognitive training and tDCS approach, including transfer to non-trained domains in participants with SCD and MCI. Clinically, these results may help develop community-based therapeutic interventions to delay or even halt cognitive decline in prodromal AD. Scientifically, these results will help elucidate individual predictors of this intervention.

## Trial status

The recruitment of participants started in May 2019. The last follow-up is expected for July 2022.

## Data Availability

Anonymized data will be made available to the scientific community upon request.

## Abbreviations

AD: Alzheimer’s disease
AEs: Adverse events
ANCOVA: Analysis of covariance
APO E: Apolipoprotein E
AVLT: Auditory verbal learning test
BDNF: Brain-derived neurotropic factor
CERAD: Consortium to Establish a Registry for Alzheimer’s Disease
CSF: Cerebrospinal fluid
DLPFC: Dorsolateral prefrontal cortex
DTI: Diffusion tensor imaging
ITT: Intention to treat
MCI: Mild cognitive impairment
MRI: Magnetic resonance imaging
NIBS: Non-invasive brain stimulation
SAEs: Serious adverse events
SCD: Subjective cognitive decline
SD: Standard deviation
tDCS: Transcranial direct current stimulation
WMT: Wiener matrices test
